# The Long Road to the Left Main: A Multidisciplinary Approach to the Revascularization of Complex Left Main Coronary Artery Disease

**DOI:** 10.7759/cureus.2281

**Published:** 2018-03-06

**Authors:** Scott Donald, Hilary Bews, Chantal Asselin, Basem Elbarouni, David Allen, Malek Kass, Siuchan Sookhoo, Davinder S Jassal

**Affiliations:** 1 Section of Cardiology, Department of Internal Medicine, University of Manitoba, Canada; 2 Physiology and Pathophysiology, University of Manitoba, Canada; 3 Section of Cardiology, Department of Internal Medicine, University of Manitoba, Canada; 4 Section of Cardiology, Department of Internal Medicine, University of Manitoba; 5 Department of Radiology, University of Manitoba, Canada; 6 Department of Internal Medicine, University of Manitoba

**Keywords:** coronary angiography, peripheral vascular disease, aortic valvuloplasty, mechanical circulatory support

## Abstract

Severe aortic stenosis (AS) is considered a contraindication to the use of mechanical circulatory support (MCS) devices, including the Impella heart pump (Abiomed, Aachen, Germany). We describe a case in which a 72-year-old female with severe AS and peripheral vascular disease (PVD) presented with retractable ischemia in the setting of a non-ST elevation myocardial infarction (NSTEMI). Using a coordinated multidisciplinary approach, our case is the first to combine iliac angioplasty, balloon aortic valvuloplasty (BAV), and the insertion of an Impella CP device in the setting of severe AS to facilitate successful coronary artery revascularization in a non-surgical patient.

## Introduction

Severe aortic stenosis (AS) is considered a contraindication to the insertion of mechanical circulatory support (MCS) devices, including the Impella heart pump (Abiomed, Aachen, Germany) [1]. To date, this notion has been challenged within the recent literature as well as within the current case report [1-7]. We present a case in which a multidisciplinary approach facilitated complex percutaneous coronary intervention (PCI) in a patient with left main coronary artery (LMCA) disease, severe AS, and bilateral iliac artery stenosis.

## Case presentation

**Figure 1:**
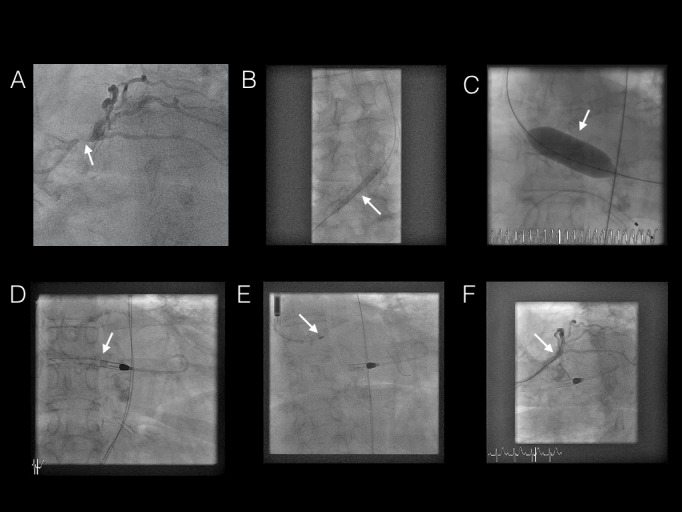
A-F A) Diagnostic coronary angiography demonstrating severe stenosis of the left main coronary artery in the left anterior oblique caudal view; B) Right common iliac balloon angioplasty; C) Balloon aortic valvuloplasty with concurrent rapid ventricular pacing; D) Impella CP catheter (Abiomed, Aachen, Germany) in situ after crossing the calcified aortic valve; E) Rotablation with a 1.5 mm burr to proximal left main coronary stenosis; F) Post-procedure left coronary angiogram demonstrating successful percutaneous coronary intervention and stenting to the left main and left anterior descending artery with the Impella catheter in situ.

A 72-year-old female with a history of hypertension, type II diabetes, dyslipidemia, peripheral vascular disease (PVD), and severe chronic obstructive pulmonary disease (COPD) presented with a non-ST elevation myocardial infarction (NSTEMI). Transthoracic echocardiography confirmed mild left ventricular (LV) systolic dysfunction with an ejection fraction of 40%-45% and severe AS with a valve area of 0.6 cm^2^. A diagnostic coronary angiography revealed a heavily calcified left main coronary artery (LMCA) with 90% stenosis, ostial left anterior descending (LAD) artery with 80% stenosis, and a ramus intermedius with 90% ostial stenosis (Figure 1A, Video 1). An intra-aortic balloon pump (IABP) was unable to be inserted due to severe PVD, which was confirmed on computed tomographic angiography (CTA). After a review by cardiac surgery, the patient was deemed not to be a surgical candidate due to underlying COPD. As the patient developed recurrent ischemic pain with diffuse ST depression across the precordial leads, a multidisciplinary approach with support from interventional radiology, structural cardiology, and interventional cardiology was pursued. Femoral balloon angioplasty to the common iliac artery was performed to facilitate the insertion of an Impella CP device, given the underlying PVD in this patient (Figure 1B, Video 2). Using a 14F short catheter and subsequently upgrading to a 14F Cook sheath, balloon aortic valvuloplasty (BAV) was performed to allow the insertion of the Impella device into the LV cavity (Figure 1C, Figure 1D, Videos 3-4). Subsequent rotablation and stenting of the left main and LAD with Resolute-Integrity drug-eluting stents was performed via radial artery access (Figure 1E, Figure 1F, Videos 5-6). Although the patient had a significant post-procedural femoral bleed requiring an interposition graft within the right femoral artery, she tolerated the procedure well and was subsequently discharged home with no recurrent ischemic symptoms at one year of follow-up.

**Video 1:** Coronary angiogram Diagnostic coronary angiography demonstrating severe stenosis of the left main coronary artery in the left anterior oblique caudal view.

**Video 2:** Peripheral vascular angiogram Right common iliac balloon angioplasty.

**Video 3:** Balloon aortic valvuloplasty Balloon aortic valvuloplasty with concurrent rapid ventricular pacing.

**Video 4:** Mechanical circulatory support Impella CP catheter in situ after crossing the calcified aortic valve.

**Video 5:** Coronary angioplasty Rotablation with a 1.5 mm burr to the proximal left main coronary stenosis.

**Video 6:** Coronary angiogram Post-procedure left coronary angiogram demonstrating successful percutaneous coronary intervention and stenting to the left main and left anterior descending artery with the Impella catheter in situ.

## Discussion

As the aging population with multiple comorbidities increases, MCS devices are being considered to provide hemodynamic support to facilitate these complex interventions [8]. The Impella device is a rotary pump that augments cardiac output by 2.5-5 L/min using continuous non-pulsatile flow [1-2]. The Impella device has advantages over other MCS devices in that it provides greater hemodynamic support and does not require a pressure wave or electrocardiographic signal [2]. In the recent PROTECT II randomized control trial comparing Impella 2.5 and IABP in patients with severe LV systolic dysfunction undergoing high-risk PCI, the use of an Impella device demonstrated superior hemodynamic support with a reduction in major adverse cardiac events at 90 days [9].

The Impella device is deployed retrograde across the aortic valve into the LV [2]. In the setting of severe AS, there exists the theoretical concern that the Impella device may further decrease the valve area or, conversely, induce valvular incompetence and subsequent hemodynamic collapse [1]. In fact, studies investigating the use of Impella devices have traditionally excluded patients with an aortic valve area < 1.5 cm^2^ [3].

Despite these concerns, a number of recent case reports have described the successful use of Impella devices in patients with severe AS requiring BAV and/or coronary angioplasty [3-6]. A case series by Spiro et al. demonstrated the feasibility of using the Impella device in five patients with severe AS (aortic valve area < 1 cm^2^) and LV systolic dysfunction (LVEF 24±5%) undergoing complex coronary intervention [1]. In four cases, BAV was necessary to advance the Impella catheter across the stenotic AV [1]. The intervention was a bridge to transcatheter aortic valve implantation (TAVI) in two patients. Additionally, Martinez et al. described the use of Impella support during high-risk PCI, BAV, or the combination of the two procedures in 21 patients with AS (aortic valve area of 0.7±0.3 cm^2^) and LV systolic dysfunction (LVEF 22±12%) [2]. Despite the success of the percutaneous interventions in both series, the 30-day mortality rate approached 15% due to underlying medical comorbidities in the patient population [2]. Finally, Singh et al. identified 116 patients from the catheter-based ventricular assist device registry with severe symptomatic AS and a mean LVEF of 27±16% who underwent BAV and/or PCI with Impella support [7]. The Impella device was electively employed in 74% of cases, with the remainder inserted for hemodynamic instability or cardiac arrest [7]. Overall, the one-year survival rate was 90% when BAV was used as a bridge to definitive therapy, as compared to 28% when employed for symptomatic relief [7].

The present case corroborates the benefits of Impella support for high-risk PCI in the setting of severe AS [1-7]. We demonstrate the use of BAV to allow the passage of the Impella catheter across the stenotic AV. Furthermore, our case is the first to combine these techniques with femoral balloon angioplasty to facilitate the insertion of the Impella device, given the severe nature of PVD in our patient. This multidisciplinary approach offers a strategy for non-surgical patients requiring PCI in the setting of severe AS and PVD.

## Conclusions

Increasingly, complex non-surgical patients are considered for PCI for medically refractory ischemic heart disease. Our multidisciplinary approach demonstrates the safety and hemodynamic benefit of Impella support in patients with severe AS and PVD undergoing high-risk PCI.
